# Ig Constant Region Effects on Variable Region Structure and Function

**DOI:** 10.3389/fmicb.2016.00022

**Published:** 2016-02-04

**Authors:** Alena Janda, Anthony Bowen, Neil S. Greenspan, Arturo Casadevall

**Affiliations:** ^1^Department of Microbiology and Immunology, Albert Einstein College of MedicineNew York, NY, USA; ^2^Department of Pathology, Case Western Reserve UniversityCleveland, OH, USA; ^3^Department of Molecular Microbiology and Immunology, Johns Hopkins Bloomberg School of Public HealthBaltimore, MD, USA

**Keywords:** immunoglobulin, isotype, constant region, variable region, structure, function

## Abstract

The adaptive humoral immune response is responsible for the generation of antimicrobial proteins known as immunoglobulin molecules or antibodies. Immunoglobulins provide a defense system against pathogenic microbes and toxins by targeting them for removal and/or destruction. Historically, antibodies have been thought to be composed of distinct structural domains known as the variable and constant regions that are responsible for antigen binding and mediating effector functions such as opsonization and complement activation, respectively. These domains were thought to be structurally and functionally independent. Recent work has revealed however, that in some families of antibodies, the two regions can influence each other. We will discuss the body of work that led to these observations, as well as the mechanisms that have been proposed to explain how these two different antibody regions may interact in the function of antigen binding.

## Introduction

Antibodies (Abs), or immunoglobulin (Ig) molecules are antimicrobial proteins that are secreted by B lymphocytes, and serve as critical participants in the adaptive immune response. The main function of Abs is to bind foreign molecules in the serum and other bodily fluids and, in most cases, label them for removal. This occurs through a form of molecular guilt by association, involving non-covalent binding of Abs to their antigens (Ag) and to cellular Fc receptors (FcRs). This removal is mediated by a variety of mechanisms associated with Ab function such as facilitation of phagocytosis, complement activation, and Ab dependent cellular cytotoxicity.

**Figure 1:**
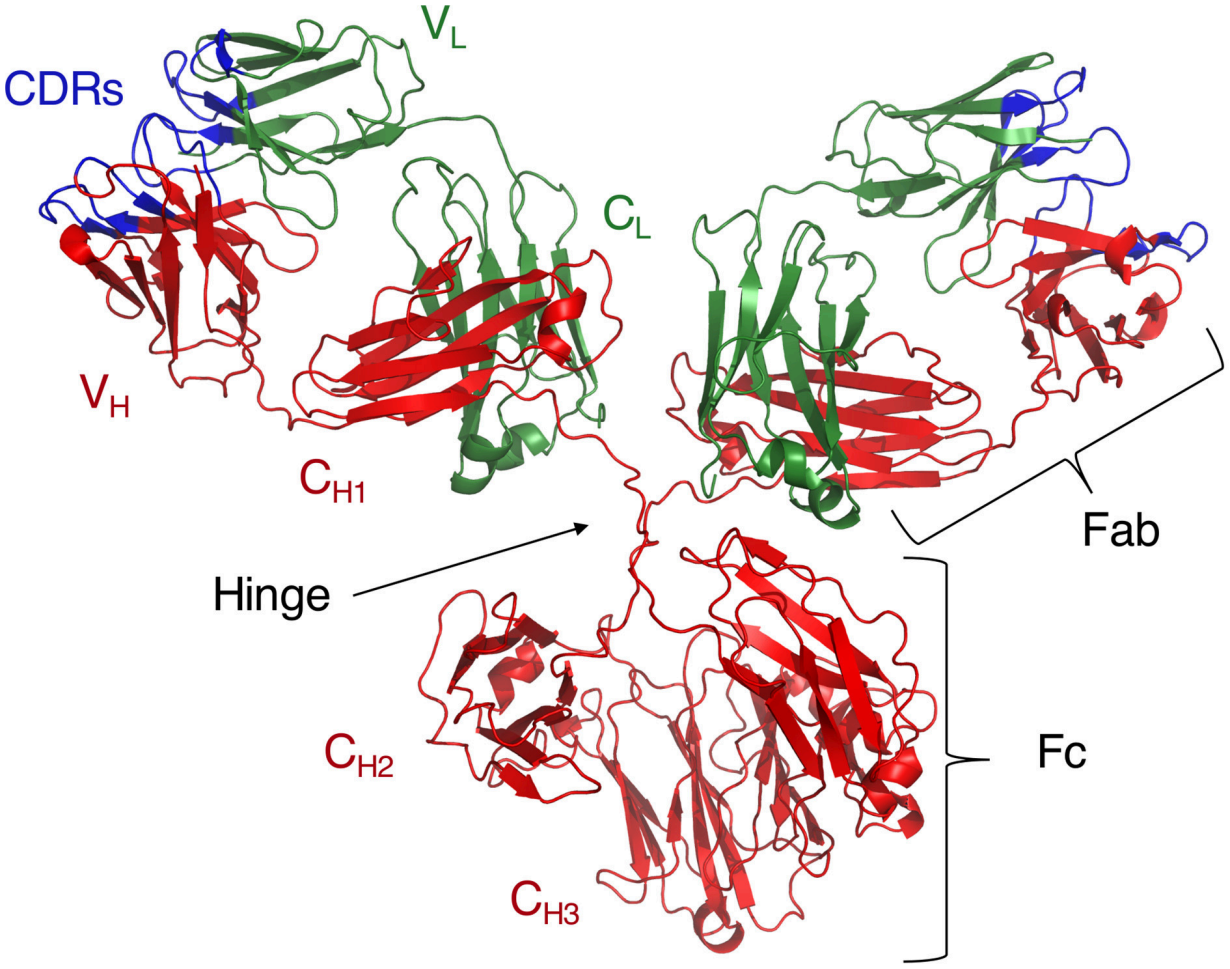
**Crystal structure of intact IgG_1_ (PDB ID: 1IGY)**. Adapted from Harris et al. (1998).

The Ig molecule consists of two polypeptide chains, a heavy (H) and a light (L) chain, each of which is composed of two regions, a constant region (C) and a variable region (V) (Figure 1). These chains form monomers which then combine into dimers, or higher-order oligomers to form a full Ig molecule. Both C and V regions contain domains from the H and L chains (Dreyer and Bennett, 1965). Functionally, the CH region confers effector properties such as complement binding, half-life length, interactions with FcRs, and the class, or isotype, of the Ig. In both humans and mice, there are four IgG, or γ-chain isotypes that are important for the identification and clearance of many peptide and polysaccharide Ags (Tonegawa, 1983). In contrast, the V region confers specificity to the Ig molecule by functioning as the direct contact between the Ig and its Ags.

## Background

Since the mid-twentieth century, the Ig molecule has been considered a bifunctional molecule consisting of two largely independent regions, a V region responsible for specificity and affinity, and a C region responsible for effector functions such as complement activation and interaction with FcRs. This view emerged from biochemical studies in the late 1950s, when Nobel laureate Rodney R. Porter used proteolytic digestion to cleave Ab molecules into fragments. These fragments eventually became known as the Ag binding fragment, or Fab, and the Fc fragment, because it could be easily crystallized (Porter, 1957; Porter and Press, 1957). One decade later, as the protein crystallography field progressed, X-ray studies of Fab fragments provided structural evidence consistent with the presumed functional separation of each adjacent V and C domain on both the H and L chain. For example, V_L_ domains were found to be separated from their C_H1_ domain neighbors by long, disordered polypeptide chains that were interpreted as spacers both physically and functionally (Davies et al., 1975). This observation was confirmed by additional studies in the 1990's (Harris et al., 1997). The disordered nature of the spacers was interpreted as inconsistent with a tight structural connection where the C influenced the structure of the V and vice versa.

## Structural and biochemical evidence that suggests two independent domains

The early hypotheses that the Fab region acted independently from the whole Ig were based on X-ray crystallographic studies of Fab and Fc fragments, Fab-hapten studies, and electron microscopy (EM) analysis of Ab–Ag complexes. Hapten studies provided additional support for the notion of two independent Ig regions as no evidence was found for structural changes in Fab molecules upon hapten binding (Stryer and Griffith, 1965; Stryer, 1968; Yguerabide et al., 1970; Werner et al., 1972; Segal et al., 1974; Harris et al., 1997). EM studies also failed to show differences in Fab tertiary structure whether this region was bound to Ag alone or as part of a whole IgG (Feinstein and Rowe, 1965).

Furthermore, the presence of hypervariable regions, or complementarity determining regions (CDRs) in the V and not the C domains suggested the evolutionary importance of the specific conformation of each V region in Ag binding (Stryer and Griffith, 1965; Stryer, 1968; Yguerabide et al., 1970; Werner et al., 1972; Segal et al., 1974; Kabat et al., 1977; Chothia et al., 1989; Harris et al., 1997). Then, in the late 1970s, Tonegawa and collaborators showed that distinct genes encoding V and C regions were rearranged and combined to express the Ig molecule, providing a mechanism for the generation of Ab diversity while maintaining Ig effector function (Tonegawa, 1983). While V regions were shown to diversify during their adaptation to specific Ags, C regions maintained their structure, supporting the hypothesis that these two regions were functionally independent.

In the early 1980s, fluorescence studies by Oi et al. failed to show an interaction between Ig V and C regions upon Ag binding (Oi et al., 1984). This negative evidence was nevertheless fully consistent with the evolving view of two independent domains. More recently, X-ray crystallographic studies of intact Igs have revealed a highly flexible hinge region described as a “loose tether” separating the Fab and Fc regions, allowing them to move freely with respect to one another (Saphire et al., 2002). We note that the classical view of two independent Ig domains emerged largely from the interpretation of negative data from a variety of structural techniques. However, there was also a certain elegance to the notion of two domains, one focused on binding and the other on downstream function, which appealed to the reductionist tenor of the times.

## Early challenges to the model

Ab–Ag complexes are readily ingested by FcR-bearing cells in contrast to the minimal ingestion of free Ab. This observation raised the possibility that the greater affinity of Ab–Ag complexes for FcRs was the result of a structural change in the Ab molecule upon binding Ag. In 1976, Huber et al. proposed an allosteric Ig model whereby Ag binding triggered a signal that traveled from the V to the C regions (Huber et al., 1976). This signal was thought to run from the V domain along conserved residues involved in inter-chain and inter-domain contacts to reach the C domain and cause a structural change that could enhance affinity for FcRs. In their final model, based on X-ray crystallographic data as well as molecular dynamics simulations of a full IgG molecule, they emphasized that all longitudinal contacts were closed. They stated that this resulted in a rigid structure where Fab_2_ bending was inhibited by C_H1_–C_H2_ interactions. They further suggested that Ag binding caused overall Ig stiffening. This stiffening was thought to occur because the flexible hinge region allowed for their hypothesized C_H1_–C_H2_ contacts, resulting in a rigid, T-shaped, Ig (Huber et al., 1976). These inferences suggested that C_H1_–C_H2_ contacts exist and are critical to Ag binding. They based their modeling studies of X-ray crystallographic IgG data on the hypotheses that Igs had both segmental rigidity and overall flexibility, and that free Fab molecules were rigid (Huber et al., 1976). These inferences were in turn based on the earlier Fab-hapten binding studies (Stryer and Griffith, 1965; Stryer, 1968; Yguerabide et al., 1970; Werner et al., 1972; Segal et al., 1974).

During this time it was also suggested that Ag binding may result in Ab conformational changes that lead to complex formation, which are in turn readily identified by macrophage FcR for uptake (Shinomiya and Koyama, 1976). This view was based on older evidence that Ab–Ag complexes were cleared from the serum at much faster rates than free Ab (Benacerraf et al., 1959). However, the view of an allosteric change in the Ab molecule that facilitated the interaction with FcR did not gain favor and was supplanted by the explanation that the greater affinity of Ag–Ab complexes was due to cross-linking of FcRs. Again, the allosteric view was abandoned largely due to negative data, while cross-linking of FcRs was a demonstrable effect that triggered signal transduction and explained increased binding of Ag–Ab complexes on the basis of increased avidity without the necessity of conformational changes.

By the 1990's the notion that Ig molecules comprised two independent non-interacting domains had largely taken root and become immunological dogma. However, several studies reported observations that seemed at odds with the hypothesis of one molecule with two independent functional regions. Over the past decade a new notion has emerged that views both Ig regions as interacting and functionally connected. These observations have also shown that V–C interactions may vary between isotype families, suggesting that the basic model of Ig structure–function needs to be reconsidered and revised.

## Isotype switching generates variants with different properties

Beginning in the early 1990s and continuing into the first decade of the twenty-first century, at least eight independent groups have reported that isotype switching is associated with altered specificity despite conservation of V region sequence (Kato et al., 1991; Pritsch et al., 1996; McLean et al., 2002; Torres et al., 2005, 2007a; Dam et al., 2008; Torres and Casadevall, 2008; Casadevall and Janda, 2012; Tudor et al., 2012; Xia et al., 2012; Hovenden et al., 2013; Dodev et al., 2015). This effect has been reported for IgM, IgG, IgE and IgA with both protein and nucleic acid Ags, using a variety of techniques such as ELISA, ITC, and SPR (Table 1).

**Table 1:** **Summary of studies finding mAb isotype differences in Ag binding**.

**Year**	**Finding**	**References**
1991	When C_L_ and C_H1_ residues were substituted in mIgG_1_, 2a, and 2b Fab isotypes, differences in Ag binding using HSQC NMR were seen.	Kato et al., 1991
1993	Murine IgG_3_ isotype was shown to self-aggregate through non-covalent Fc–Fc interactions, thereby causing differences in affinity for and binding to multivalent Ags as compared to its IgG_1_ isotype.	Greenspan and Cooper, 1993
1996	hIgA1, hIgG_1_, hIgG_2_, and hIgG_4_ isotypes were found to have different KD constants using SPR.	Pritsch et al., 2000
2002	mIgG_1_, and mouse-human chimeras (murine V-region with human constant regions 1, 2, 3, 4, M, and A1) were seen to have different Ag binding profiles by ELISA and Immunofluorescence.	McLean et al., 2002; Torres et al., 2007a
	SPR studies showed no changes in thermodynamics upon deglycosylation of mIgG_1_, as well as significant differences between mIgG1 and its chimera.	
2005–2015	mIgG_1_, 2a, 2b, and 3 isotypes were shown to have different Ag binding profiles and kinetics using ELISA, Immunofluorescence, ITC and SPR. They were further found to have differences in Trp fluorescence, CD spectroscopy with multivalent Ag, and chemical shifts by NMR when binding to a monovalent Ag. Monovalent peptide Ag cleavage assays revealed different abilities to cleave Ag as well as different rates of cleavage between the isotypes that cleaved.	Torres et al., 2005, 2007b; Janda and Casadevall, 2010; Casadevall and Janda, 2012; Janda et al., 2012, 2015; Eryilmaz et al., 2013
2012	A human IgG_1_ and its IgGA2 isotype were found to have differences in HIV-1 gp41 epitopes as well as Ag affinities measured by ELISA.	Tudor et al., 2012
2012–2013	mIgG_1_, 2a, 2b, and 3 isotypes displayed differences in affinity to the same Ag using SPR methods, as well as isotype differences in Trp fluorescence and CD spectroscopy upon Ag binding.	Xia et al., 2012, 2013
2013	mIgG_1_, 2a, 2b, and 3 isotypes were found to have different affinities to Ag using SPR.	Hovenden et al., 2013
2013	Human IgG_1_ and IgA2 found to have different affinities for HIV-1 Env Ag by SPR.	Tomaras et al., 2013
2015	Human IgG, IgA, IgE isotypes compared by SPR, found to have different Ag affinities.	Dodev et al., 2015

An early study from Kato et al., found significant changes in Ag binding when comparing murine isotype Fabs with identical V_L_, C_L_, and V_H_ sequences as well as an Fab with a complete C_H1_ domain deletion mutant (Kato et al., 1991). They used ^13^C-Nuclear Magnetic Resonance (NMR) as a spectroscopic probe of both lateral and longitudinal domain-domain Fab interactions upon Ag binding in a group of murine Fabs derived from IgG_1_, IgG_2a_, and IgG_2b_ isotypes. They were also able to probe chemical shifts directly responsible for Ag binding within the CDR3 loop. They found isotype differences in chemical shifts in the CDR3 region, or paratope as well as other, non-Ag binding residues throughout the Fabs. This work implied that subtle chemical shifts in both the paratope and other, more distant residues occurred within Fab molecules upon Ag binding, and that these shifts could vary depending on isotype (Kato et al., 1991).

Another study, which appeared shortly afterward, used human monomeric Fabs derived from IgA_1_ and IgG_1_ in Surface Plasmon Resonance (SPR) experiments with a monovalent Ag. Significant differences in Ab–Ag association rate constants between the Fabs studied were found (Pritsch et al., 1996). In this work the authors suggested that in a particular mAb, affinity maturation could be achieved through class switching, suggesting a role for the C_H1_ domain in allosterically influencing the Ag binding site (Pritsch et al., 1996). The same group, in 2000, isolated four human IgG mAbs, three of which had identical V_L_ and V_H_ domains. Among this set, the IgA_1_ and IgG_1_ isotypes manifested significant differences in affinity, though they bound to the same Ag motif. They then created IgG_1_ and IgM from the IgA_1_ mAb, and found that the IgM bound to its epitope with the same affinity as the parental IgA_1_, whereas the IgG_1_ had significantly lower affinity as measured by SPR. This result was reproducible using Fabs derived from IgG_1_ and IgA_1_ (Pritsch et al., 2000). Consequently, they hypothesized that conformational changes in Fab molecules were occurring upon Ag binding, and were most likely being transferred through the elbow angle. Furthermore, Pritsch et al. were able to create a model of the V_H_–C_H1_ interface of their IgA_1_ and IgG_1_ using X-ray crystallographic structures derived from homologous proteins, and found that one of the C_H1_ loops was directly involved in V_H_–C_H1_ contacts. Their model also showed that this loop had a different conformation for each isotype (Pritsch et al., 2000). This work supports the hypothesis that the C_H1_ domain has an allosteric role in Ag binding and may also be transmitting structural signals to the rest of the Ig as suggested by Huber et al.'s earlier work.

McLean et al. made chimeric mAb isotypes (murine V region with human IgA_1_, IgG 1/2/3/4, and IgM C regions) that showed differences in fine specificity from the parent murine Ab. Furthermore, they saw differences between the various chimeric Abs upon binding monovalent and multivalent Ags, as well as differences in their binding location to a microbial capsule (McLean et al., 2002). Further SPR studies using this family of mAbs compared the intact murine IgG_1_ with its chimeric form as well as its deglycosylated form. These studies revealed that glycosylation did not alter Ag binding kinetics and there were no significant differences in Ag binding between the original mAb and its murine–human chimeric form (Torres et al., 2007a). However, it is unclear whether C region glycosylation may play a role in the Ag binding of other families of isotypes, as this has not been thoroughly studied.

A series of studies with the 3E5 family of murine isotypes to *Cryptococcus neoformans* polysaccharide also identified significant changes in isotype specificities and affinities. Using both multi and monovalent Ags, and ELISA, SPR, and Isothermal Titration Calorimetry (ITC) studies, the authors measured significant differences in binding among the four IgG isotypes (Torres et al., 2005, 2007b; Dam et al., 2008). A monovalent peptide Ag mimetic was used for ELISA binding studies, SPR, and ITC studies. SPR was done with Fabs derived from the 3E5 murine IgG isotype set to isolate the V region and a single C region—the C_H1_. Among the isotypes, the IgG_1_ Fab showed the most favorable binding parameters. This implied that differences in specificity among the isotypes were potentially due to differences in the C_H1_ region alone (Torres et al., 2007b). ITC studies done with the 3E5 family using full IgG molecules and the P1 peptide Ag, confirmed a 2:1 binding stoichiometry of peptide:Ab as well as significantly different association constants between all four isotypes (Dam et al., 2008).

In 2012, Tudor et al. reported increased monovalent Ag specificity and binding affinity when switching an anti-HIV-1 human IgG_1_ to a monomeric IgA2. They also found altered epitope specificity and increases in anti-HIV-1 activity assays, indicating significant changes to the Ig paratope (Tudor et al., 2012). A study by Crespillo et al., with the same family of mAbs, compared ITC binding parameters between the Fab and whole IgG of human 2F5. Their results showed significant differences in binding affinities between the different forms of Ab and monomeric peptide Ag epitopes, with highest affinities achieved with whole IgG (Crespillo et al., 2014). This adds to the observation that in this case, in addition to the C_H1_ region, the C_H2_ and/or C_H3_ regions may also be playing a strong role in Ag binding.

More recently, Xia et al. expanded this observation to include anti-nuclear mAbs. They discovered that a family of anti-DNA murine mAb IgG isotypes had significant differences in binding affinities by SPR. They also studied Trp fluorescence and circular dichroism with these isotypes and observed changes upon Ag binding that were isotype dependent (Xia et al., 2013).

For a set of Abs binding to the *Bacillus anthracis* capsule class-switching from the original IgG_3_ to IgG_1_, IgG_2a_, and IgG_2b_ isotypes resulted in a loss of protection, affinity and a change in mAb binding to its capsular Ag (Hovenden et al., 2013). Hovenden et al. further determined that switching the C_H1_ region of the protective isotype with highest affinity (IgG_3_), for the C_H1_ region from a non-protective isotype with the lowest affinity (IgG_2b_), showed no loss of affinity or protection. However, swapping the C_H2_ or C_H3_ regions from the IgG_2b_ to the IgG_3_, reduced affinity and resulted in a loss of protection, more so with the C_H2_ region (Hovenden et al., 2013). This contrasts with earlier studies that reported similar binding differences between Fab fragments and whole IgG, suggesting an allosteric role for the C_H1_ domain (Yuan et al., 1995, 1998; Torres et al., 2007b). Furthermore, Hovenden et al. used a monovalent peptide to measure fluorescence perturbation as an indication of intrinsic affinity, and found significant differences among the isotypes. Although they could not exclude a contribution from Fc–Fc interactions, there was evidence that other factors must also be contributing to the observed changes in affinity (Hovenden et al., 2013). Using their family of isotypes, they were also able to exclude the hypothesis that as flexibility of the hinge region increases, Ag binding affinity increases. This hypothesis had been suggested by Morelock et al. to explain how isotype could influence human mAb affinity (Morelock et al., 1994; Tomaras et al., 2013). Hovenden et al. further hypothesized that the contributions from the C region in this family of mAb isotypes may be due to (i) glycosylation, although de-glycosylation of their IgG_3_ did not change its functional affinity, (ii) possible C_H2_ influence on mAb charge, or (iii) C region effects on the chemical and/or electrostatic environment of the paratope. These studies shed light on the different behaviors of different families of mAb isotypes and the need to further investigate and understand how C regions affect paratope properties and how this may differ between different mAb families.

Recently, several studies have identified additional instances where Abs with identical V regions differing in isotype manifest differences in Ag binding. A group studying human anti-HIV-1 Env mAbs identified that an IgA_2_ and IgG_1_ sharing the same V region had significantly different Env Ag binding affinities using SPR studies. In fact, they found that the IgA_2_ bound one Ag strain with 2.9-fold higher magnitude and affinity and another Ag strain with 40-fold greater affinity than the IgG_1_ isotype (Tomaras et al., 2013). Another group studying the Phl p7 grass pollen allergen generated a full panel of human IgG and IgA isotypes and found subtle but significant differences in binding rates using SPR. They observed the greatest differences in both on- and off-rate constants to be about three-fold, which canceled out to give an overall affinity range of 250–570 picomoles (pM) (Dodev et al., 2015).

**Figure 2:**
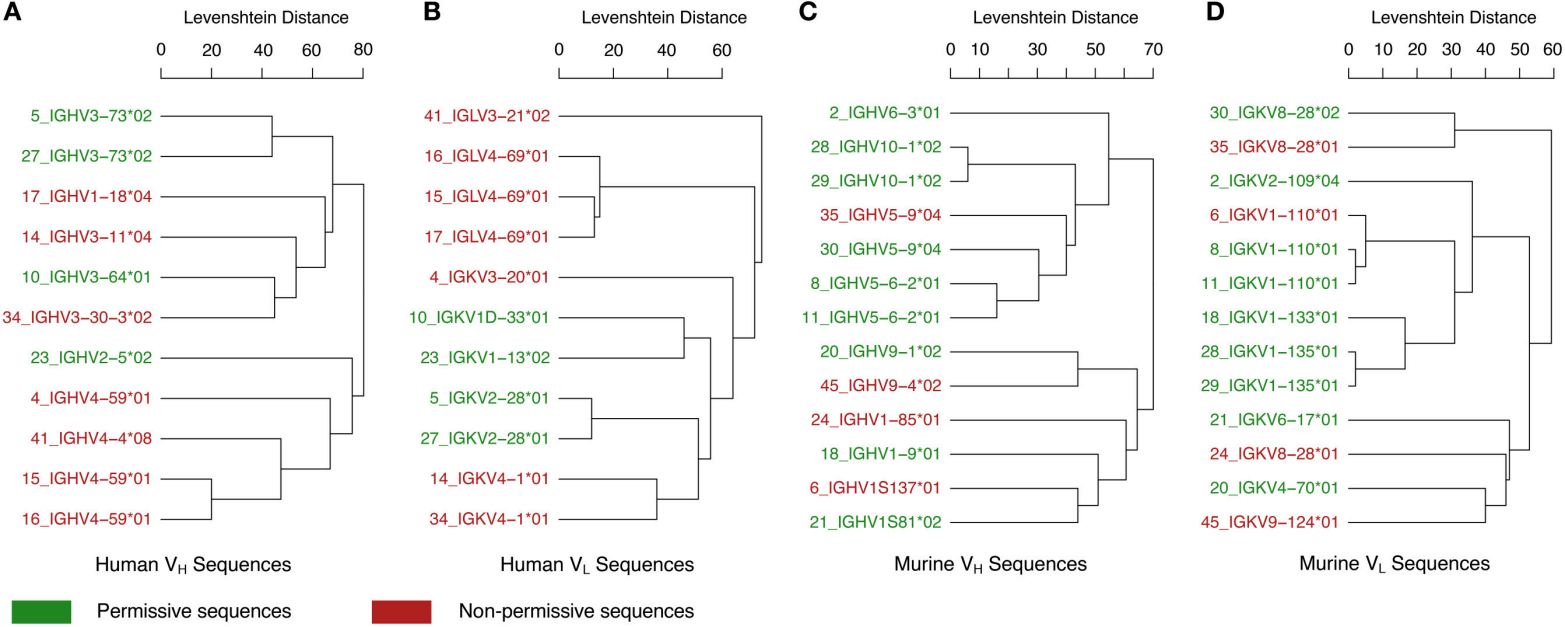
**Relationships between permissive and non-permissive V region sequences**. Immunoglobulins with differing C regions and identical V regions were identified in the literature (Table 2). VH and VL amino acid sequences were found for 24 of these unique antibodies (11 human and 13 murine, Table 2). Human sequences were grouped by **(A)** VH and **(B)** VL sequences. Murine sequences were also grouped by **(C)** VH and **(D)** VL sequences. For each group, a dendrogram was constructed through hierarchical average-linkage clustering with pairwise sequence similarity calculated as the Levenshtein distance. Leaf labels in the dendrograms are colored according to whether changes in the constant region for that antibody were permissive (green) or non-permissive (red) of specificity changes. The top V-region gene candidate for each amino acid sequence was determined with the IGBLAST blastp program using default parameters for either the human or mouse database (IGBLAST). Leaf labels in the dendrograms are of the form “seqID_top V-region gene hit,” where seqID matches the sequence ID given in Table 2.

In addition to the various examples of V region-identical Igs exhibiting specificity differences, it is important to note that several studies have reported V region-identical Igs with no changes in specificity. Although the failure to detect changes could be the result of insufficient sensitivity in the assays done, it is possible that the phenomenon of C-mediated changes in specificity and affinity is associated with some V regions and not others. Consequently we compared unique V_H_ and V_L_ sequences from 24 Igs with V region-identical isotype switch variants compiled from the literature (Table 2). An analysis of the sequence similarities and germline gene hits for these Igs shows that these appear to group phylogenetically, consistently with the notion that certain V region gene families may differ in being permissive or non-permissive of specificity changes following class switching (Figure 2). It is interesting to note that all of the human lambda V_L_ Igs were non-permissive. With the small number of Ig sequences available for this analysis, no firm conclusions can be made and more examples will be needed to confirm a V region germline basis for C domain-mediated specificity changes. It will also be useful to compare the structures of Igs with very similar V regions that differ in specificity to determine whether they are permissive of specificity changes, such as V_L_ sequences 6, 8, and 11 in Table 2. Furthermore, the combination of specific V_H_ and V_L_ genes is another potential variable for C domain-mediated specificity changes since it is conceivable that even for permissive V regions that expression of this effect requires combination with certain C regions. In this regard it is noteworthy that different C regions combined with the same V region manifested differences in the magnitude of the changes observed (Janda et al., 2015).

**Table 2:** **Unique antibodies with variable-region-identical isotype-switch variants and identifiable amino acid sequences**.

**seqID**	**Antibody**	**Organism**	**Permissive**	**Top VL gene**	**Top VH gene**	**References**
2	HGAC 39.G3, G1, G2b	Murine	Y	IGKV2-109^*^04	IGHV6-3^*^01	Greenspan and Cooper, 1993
4	F105 mAb anti-gp120 G1, G3	Human	N	IGKV3-20^*^01	IGHV4-59^*^01	Marasco et al., 1992
5	anti-tubulin mAb	Human	Y	IGKV2-28^*^01	IGHV3-73^*^02	Pritsch et al., 2000
6	IF6, 1E1, 2E12 mAbs anti-O6 LPS	Murine	N	IGKV1-110^*^01	IGHV1S137^*^01	Pollack et al., 1995
8	18B7 anti-GXM	Murine	Y	IGKV1-110^*^01	IGHV5-6-2^*^01	Mukherjee, 1992
10	F425 mAb anti-gp140 G2, G1, G3, A	Human	Y	IGKV1D-33^*^01	IGHV3-64^*^01	Liu et al., 2003
11	3E5 anti-GXM	Murine	Y	IGKV1-110^*^01	IGHV5-6-2^*^01	Mukherjee, 1992
14	F240 mAb anti-HIV G1, G3, G4	Human	N	IGKV4-1^*^01	IGHV3-11^*^04	Cavacini et al., 1998
15	F598 mAb anti-PNAG G2, G1	Human	N	IGLV4-69^*^01	IGHV4-59^*^01	Kelly-Quintos et al., 2006
16	F628 mAb anti-PNAG G2, G1	Human	N	IGLV4-69^*^01	IGHV4-59^*^01	Kelly-Quintos et al., 2006
17	F630 mAb anti-PNAG G2, G1	Human	N	IGLV4-69^*^01	IGHV1-18^*^04	Kelly-Quintos et al., 2006
18	2G8, 1E12 mAbs anti-beta glucan G2b, M	Murine	Y	IGKV1-133^*^01	IGHV1-9^*^01	Torosantucci et al., 2009
20	12.8 mAb anti-pfMSP1	Murine	Y	IGKV4-70^*^01	IGHV9-1^*^02	Porter and Press, 1957
21	12.10 mAb anti-pfMSP1	Murine	Y	IGKV6-17^*^01	IGHV1S81^*^02	Porter and Press, 1957
23	2F5 bNAb anti-HIV1 G1, A2	Human	Y	IGKV1-13^*^02	IGHV2-5^*^02	Kunert et al., 1998
24	C1 mAb anti-pfMSP1 G1, G2a, G2b, G3	Murine	N	IGKV8-28^*^01	IGHV1-85^*^01	Adame-Gallegos et al., 2012
27	IgA1 and IgG1 crystallized Fabs	Human	Y	IGKV2-28^*^01	IGHV3-73^*^02	Correa et al., 2013
28	F24F2 mAb anti-γdPGA *B. anthracis* G3, G1, G2a, G2b	Murine	Y	IGKV1-135^*^01	IGHV10-1^*^02	Kozel et al., 2007
29	F26G3 mAb anti-γdPGA *B. anthracis* G3, G1, G2a, G2b	Murine	Y	IGKV1-135^*^01	IGHV10-1^*^02	Kozel et al., 2007
30	PL9-11 mAb G3, G1, G2a, G2b	Murine	Y	IGKV8-28^*^02	IGHV5-9^*^04	Xia et al., 2013
34	FI6 bNAb anti-HA stalk G1, G2a, DA265	Human	N	IGKV4-1^*^01	IGHV3-30-3^*^02	Porter, 1957
35	PY102 mAb anti-HA head G1, G2a, DA265	Murine	N	IGKV8-28^*^01	IGHV5-9^*^04	Zaghouani et al., 1989
41	PGT121	Human	N	IGLV3-21^*^02	IGHV4-4^*^08	Walker et al., 2011
45	20B1 G1, G2a, G2b	Murine	N	IGKV9-124^*^01	IGHV9-4^*^02	French et al., 1957

These studies necessitate expanding the role for isotype class switching to a new role where it contributes to the generation of Ig diversity. A recent review by Sela-Culang et al., suggests that changes in C regions or their conformations may lead to structural C domain rearrangement relative to other C and V domains. They suggested that this could re-shape the Ag binding site and as such could be considered as a mechanism for generating Ab diversity (Sela-Culang et al., 2013). This is further supported by the hypothesis that the conformational diversity of Abs is directly linked to Ab multispecificity and supports the role of a single sequence (i.e., V region) adopting multiple structures and functions (Reitan and Hannestad, 1995, 2001; Lange et al., 1996; James and Tawfik, 2003). Since the mechanisms of isotype switching and somatic mutation share some of the same proteins this notion has the elegance of bringing together these two processes within the same molecular pathways. Isotype class switching has further implications for primary and secondary B cell responses, idiotype reactivity and immunogenicity. Also, the observation that Ig class switching can result in reactivity for self Ags despite identical V regions suggests that this phenomenon may be implicated in the appearance of certain pathological autoimmune responses (Torres et al., 2007b). An understanding of how C regions affect Ab paratope is important for the development of therapeutic mAbs (Nosanchuk, 2013). At least two mechanisms have been proposed for this effect: (i) C region-mediated allosteric effects on V region paratope structure before and/or during Ag binding (Huber et al., 1976; Janda et al., 2012) and (ii) subclass-mediated differences in functional affinity leading to the differential recognition of multivalent epitope arrays (Cooper et al., 1993). A plausible mechanism on the molecular level for the first of these effects involves C-mediated structural constraints on V region structure that affect the conformation of the Ig paratope. In contrast, C region glycosylation has thus far not been shown to contribute to this phenomenon (Torres et al., 2007a,b; Hovenden et al., 2013).

## C region modification of specificity through increased functional affinity

In 1987, Greenspan et al. reported that murine IgG_3_ mAbs specific for the cell wall polysaccharide of *Streptococcus pyogenes* were able to bind cooperatively to whole bacteria treated with a protease to make the relevant epitopes accessible. The Fc regions were found to be required for this enhanced binding. Subsequently, Cooper et al. (1991) demonstrated that IgG_3_ bound more strongly to treated (as above) group A streptococcal bacteria than IgG_1_ and IgG_2b_ switch variants confirmed to be V domain-identical by cDNA sequencing (Greenspan et al., 1987; Cooper et al., 1991).

Cooper et al. (1993) further demonstrated that in this system the IgG_3_ mAb exhibited a different pattern of binding to three strains of group A streptococci displaying the relevant epitope at different densities than the IgG_1_ or IgG_2b_ intact Ig molecules or F(ab')_2_ fragments of IgG_3_. While the IgG_1_ and IgG_2b_ Abs and the IgG_3_ F(ab')_2_ fragments bound best to the streptococcal strain with the highest epitope density, the IgG_3_ Ab bound best to the strain with intermediate epitope density. Additional experiments verified that despite these differences in binding to multivalent Ags, the three Abs (IgG_3_, IgG_1_, and IgG_2b_) bound to monovalent Ag or multiple rat anti-idiotypic mAbs comparably (Cooper et al., 1993).

Thus, in spite of identical V domain amino acid sequences and ability to bind monovalent hapten, the IgG_3_ Ab discriminated among multivalent antigens differently than the IgG_1_ or IgG_2b_ Abs (i.e., exhibited different multivalent fine specificities). Additional conclusions drawn by the authors are that epitope density and H chain C region structural differences can contribute to differences in multivalent binding among IgG subclasses. Analysis of this Ab-Ag system with SPR revealed that the stronger binding of the cooperative IgG_3_ Ab, in comparison to the non-cooperative IgG_1_ and IgG_2b_ Abs, was associated with both greater on rates and slower off rates, consistent with the hypothesis that non-covalent Fc–Fc interactions mediated the cooperativity of the IgG_3_ Ab (Cooper et al., 1994).

Also in 1993, Schreiber et al. generated an IgG_3_ isotype switch to a murine IgG_1_ mAb against a *Pseudomonas aeruginosa* polysaccharide Ag, as well as an IgG_1_-derived F(ab)_2_. These authors found that the intact IgG_3_ molecule had stronger binding affinities than the V domain identical IgG_1_ Ab to the *P. aeruginosa* Ag, and they hypothesized that this better binding was most likely due to the greater functional affinity (i.e., multivalent affinity or avidity) of the murine IgG_3_ Ab (Schreiber et al., 1993).

In roughly the same time period, Izui and colleagues published a number of papers focused on murine IgG_3_ cryoglobulins in autoimmune disease models that reported data consistent with the results of Greenspan and his associates. For example, Fulpius et al. studied a pathogenic murine IgG_3_ mAb derived from a MRL/MpJ-lpr/lpr mouse and exhibiting cryoglobulin and rheumatoid factor activity for IgG_2a_ along with a V domain-identical switch variant Ab of the IgG_1_ subclass. The IgG_1_ Ab lacked the cryoglobulin activity and displayed at least a 90% reduction in ability to bind to IgG_2a_ Fc regions (Fulpius et al., 1993).

Another group with similar results on isotype differences in avidity during this time used a set of chimeric mAbs (murine V region, human C region) against both monovalent and bivalent intercellular adhesion molecule 1. They used ELISA competition studies with whole mAb as well as chimeric and murine Fab fragments. Though full-length mAbs showed differences in competition ELISA, their murine and chimeric Fab counterparts had equivalent binding constants, indicating that any differences in whole mAb were due to differences in avidity, and not monovalent affinity (Morelock et al., 1994).

## C region modification of specificity through allosteric changes

Circular dichroism studies done on a family of murine IgGs to *C. neoformans* polysaccharide showed that the C and V regions are structurally coupled and affect each other during Ag binding (Janda and Casadevall, 2010). This was followed by tryptophan fluorescence studies of the same family of murine IgG Abs which showed different changes in electrical properties of Ig Fab Trp molecules some of which are in the paratope, upon Ag binding. NMR studies of the same group of Igs further expanded the notion by showing differences in the chemical environments of their paratopes, as well as IgE and IgA isotypes. Finally, X-ray crystallographic studies and molecular modeling of these Igs identified structural differences that occur mainly in the hinge angles among Fab molecules of the IgG_1_ and IgG_3_ isotypes (Janda et al., 2012, 2015).

In 2003 Adachi et al. used molecular dynamics simulations to compare the crystal structures of a murine mAb IgG_1_ Fab alone, Fab in complex with its hen egg lysozyme (HEL) Ag and the Fv (V regions only) in complex with HEL (Adachi et al., 2003). The Fv–HEL complex was found to have a dissociation constant one order of magnitude lower than that of the Fab–HEL complex (Lavoie et al., 1992). In addition, Adachi et al. identified significant differences in the structures including (i) 18 water molecules in Fv-HEL interface, while the Fab-HEL interface had only one, (ii) the second upper loop in the C_L_ domain in the Fab light chain (UL2-C_L_) showed large conformational fluctuations when compared to the crystal structure of Fab alone. The difference in water molecules in the interface may represent a much tighter interaction for the Fab-HEL complex, which may be essential for HEL binding and could explain the differences in dissociation factors. The authors hypothesized that removal of the C domains in the Fv molecule may result in imperfect complementarity between Ab and Ag, and thus lead to an increase in water molecules between paratope and epitope (Adachi et al., 2003). Furthermore, the UL2-C_L_ region, which is highly conserved in human and murine light chains, was previously predicted to have unique fluctuations corresponding with Ag binding (Kabat et al., 1975). These new studies indicate that the UL2–C_L_ fluctuations may be playing an important role in allosteric mediation of paratope–epitope interactions (Adachi et al., 2003).

In 2013, Xia et al. found that anti-DNA mAb V-domain identical switch variants from a murine SLE autoantibody model have different changes in secondary structure upon Ag binding, confirming coupling of the C and V regions. In addition, they found significant differences in histone and kidney Ag binding profiles using SPR, and different changes in Trp fluorescence upon Ag binding. Moreover, these differences were associated with significant differences in renal pathogenicity and survival studies that included *in-vivo* Ab administration (Xia et al., 2013).

X-ray crystallographic studies of V region identical human Fab from IgA_1_ and IgG_1_ by Correa et al. reveal greater rigidity in the C_H1_–C_L_ and C_H1_–V_H_ interfaces in the IgA_1_ structure, suggesting that these could exert allosteric effects on the paratope. They identified a large hydrophobic core of residues in the IgA1 V_H_–C_H1_ interface, as well as a disulfide bridge connecting heavy and light chains which was absent in the IgG_1_ model. Furthermore, they identified a difference of about 5° in the angle between the V_H_–C_H1_ domains of the two Fabs which modifies the V_H_–V_L_ arrangement. This subtle change may result in allosteric effects that lead to critical rearrangements of the paratope. These constraints ultimately led to an increased rigidity of the IgA1 molecule and different conformational entropy that is hypothesized to correlate to Ag binding affinity modulation (Correa et al., 2013).

A comparison of human and murine mAb IgG isotypes using the full ensemble optimization method and SAXS data of these isotypes by two separate groups revealed important conformational differences (Eryilmaz et al., 2013; Tian et al., 2015). Eryilmaz et al. proposed that differences in global structures of isotypes are due to cross-domain relationships between various V and C region combinations. They hypothesized that these relationships can dramatically change the overall shape of an Ig (Eryilmaz et al., 2013). This was supported by studies by Tian et al. which showed that differences seen in Ag binding may be related to intermolecular Fab–Fab and Fab–Fc interactions. This was based on the significant differences in hinge angles and the type of overall conformation that was adopted by the IgG molecules they used (Tian et al., 2015).

Recent studies comparing 141 crystal structures of Abs with and without Ag provided additional insight into C region involvement in Ag binding. These studies show that Ag binding is associated with changes in (1) H and L chain relative orientations in both C and V domains, (2) elbow angles between V and C regions, especially when binding large Ags, and (3) C_H1_ loops implicated in interactions between H and L chains which show the most consistent and substantial changes upon Ag binding. Although the group only compared pairs of structures solved with the same space group, no two pairs had similar unit cell sizes. However, these results were consistent among many pairs of Fabs and their Fab-Ag structures, and provide some insight into how C-region mediated allosteric effects could potentially transmit to the V region (Sela-Culang et al., 2013).

## Antigen-induced changes in Fc region

It is also worth mentioning that there are a few studies that have hypothesized allosteric changes in Fc regions upon Ag binding, thus promoting Ab effector functions. This strengthens the argument for Ig inter-molecular signaling through specific structural interactions. For example, in 1970's Brown and Koshland identified changes in J-chain exposure in C_H4_ Fc regions of IgM molecules that were directly induced by monovalent Ag binding to the Fab region. Their studies excluded cross-linked Ags (Brown and Koshland, 1975, 1977). In addition, studies by Schlessinger et al. using Trp fluorescence and monovalent Ags with anti-RNase Abs demonstrated significantly different C-region changes in both Fab and whole Ab upon Ag binding. This suggests an interaction between Ag binding sites and distant Fc regions. When they reduced the inter-chain disulfide bonds of the Fabs and whole Abs, they no longer saw these changes in whole Ab molecules. This work indicates that disulfide bonding especially at the Ab hinge region is required for the transmission of allosteric signaling from the Fab region to the Fc in these Ab molecules (Schlessinger et al., 1975). Another study, which assessed IgG Fc binding to staphylococcal protein A and streptococcal protein G proteins (which bind to Ig C domains), identified inhibition of binding in the presence of Ag, presumably through changes to the Fc region. These observations were also made in the presence of a reducing environment (Oda et al., 2003). These studies raise the question of Ig allosteric effects that occur after Ag binding and travel down the molecule to induce changes in the Ig Fc region.

## Concluding remarks

Since the initial findings that Ig C regions can change the Ag binding parameters of their V regions, more recent studies have begun to attempt to elucidate the allosteric mechanisms through which these effects may be occurring. Many studies have now shown that inter-molecular interactions between heavy and light chains, V and C regions, hinges and elbow relationships, all can play a vital role in the overall molecular structure of the Ig paratope in relationship to its affinity and specificity for Ag. In particular, the C_H1_ region seems to play a large role in determining Ag binding parameters, most likely through its intermolecular interactions with both its neighboring C_L_ domain as well as by transmitting structural information to the Ig hinge region and back to the V_H_ domain.

## Author contributions

AJ wrote the manuscript, Table 1, and edited the document to prepare for submission. AB put together Figures 1, 2, and Table 2 and also edited the paper. NG contributed with writing and editing the review. AC is the corresponding author, he wrote and edited the review.

## Funding

AC was supported by NIH awards HL059842, AI033774, AI052733, AI033142. AJ wishes to acknowledge support from the Institutional AIDS training grant T32-AI007501 and the MSTP training grant, T32-GM007288. AB wishes to acknowledge support from the MSTP training grant: T32-GM007288. Authors AC, AJ and AB would also like to acknowledge funding from Albert Einstein College of Medicine, Yeshiva University.

### Conflict of interest statement

The authors declare that the research was conducted in the absence of any commercial or financial relationships that could be construed as a potential conflict of interest.

## Abbreviations

Ig: immunoglobulin
Ab: antibody
Ag: antigen
mAb: monoclonal antibody
CH: heavy chain constant region
CL: light chain constant region
Fab: antigen binding fragment
Fc: crystallizable fragment
SPR: Surface Plasmon Resonance
ITC: Isothermal Calorimetry
ELISA: Enzyme-Linked Immunosorbent Assay.
